# Abdominal Wall Abscess Caused by Small Intestinal Penetration That Was Difficult to Distinguish from a Malignant Tumor: A Case Report

**DOI:** 10.70352/scrj.cr.25-0251

**Published:** 2025-07-19

**Authors:** Tomoya Kurose, Shoichi Inokuchi, Satoshi Tsutsumi, Takahiro Terashi, Masahiko Ikebe, Toshio Bandoh, Tohru Utsunomiya

**Affiliations:** Department of Surgery, Oita Prefectural Hospital, Oita, Oita, Japan

**Keywords:** abdominal wall abscess, fish bone, foreign body ingestion, laparoscopic-assisted surgery

## Abstract

**INTRODUCTION:**

Fish bone ingestion is common but rarely causes complications such as abdominal wall abscesses, which can mimic malignancies such as sarcomas on imaging. Abscesses require drainage and antibiotics, while sarcomas need wide excision. Therefore, the differentiation between abscesses and sarcomas is important and often requires multidisciplinary involvement.

**CASE PRESENTATION:**

A 70-year-old woman presented with anorexia and a painful abdominal mass. Laboratory tests showed inflammation but normal tumor marker concentrations. The abdominal wall mass was hard and poorly mobile. Ultrasound showed a heterogeneous, mosaic-like internal structure, and CT and positron emission tomography-CT findings strongly suggested a malignant tumor such as sarcoma. We performed surgery and confirmed the presence of an abdominal wall abscess with small intestinal penetration caused by an ingested fish bone. The small intestine was partially resected, and pathology showed no malignancy. The patient recovered well and was discharged on postoperative day 9. The final diagnosis was an abdominal wall abscess caused by an ingested fish bone that perforated the small intestine.

**CONCLUSIONS:**

We present a rare case of an abdominal wall abscess caused by penetration of the small intestine by an ingested fish bone.

## Abbreviations

CRP
: C-reactive protein
CT
: computed tomography
MRI
: magnetic resonance imaging
WBC
: white blood cell

## INTRODUCTION

Fish bone ingestion is common and typically harmless, but rare complications such as gastrointestinal perforation can lead to abdominal wall abscesses.^1,2)^ These abscesses are difficult to diagnose, as they can mimic malignancies such as sarcomas due to overlapping clinical symptoms and imaging findings. On imaging, abscesses typically appear as thick-walled fluid collections with ring enhancement, while sarcomas present as heterogeneous soft-tissue masses with invasive features. The diagnosis is further complicated when patients do not recall ingesting a foreign body.^3)^ However, accurate differentiation between the 2 conditions is important because abscesses are treated with drainage and antibiotics, while sarcomas need wide surgical resection and reconstruction, often requiring multidisciplinary involvement.

In the present report, we describe a patient with an abdominal wall abscess caused by an ingested fish bone that penetrated the small intestine.

## CASE PRESENTATION

**Fig. 1:**
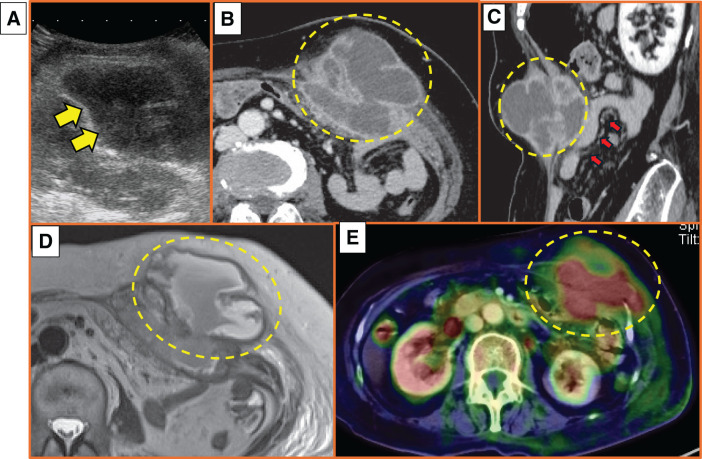
Imaging examinations at the initial visit. (**A**) Ultrasound shows a heterogeneous, mosaic-like internal structure (yellow arrows). (**B**) Enhanced CT (horizontal) shows an 8-cm low-density tumor in the left side of the abdominal wall (yellow dotted circle). (**C**) Enhanced CT (sagittal) shows that the tumor is in contact with the small intestine (yellow dotted circle; red arrows indicate the small intestine). (**D**) Dynamic MRI shows that the tumor has high signal intensity on T2-weighted imaging (yellow dotted circle). (**E**) Positron emission tomography-CT shows fluorodeoxyglucose uptake corresponding to the solid component of the tumor. The mass was suspected to have spread into the abdominal cavity, a finding suggestive of malignancy (yellow dotted circle).

A 70-year-old woman presented to our hospital with anorexia. She had a history of diabetes mellitus, hypertension, hyperlipidemia, and appendectomy. She had no history of alcohol use or smoking. Physical findings revealed a fist-sized palpable mass on the left side of the abdomen and pain in the same area. The mass was hard and poorly mobile. She did not have a fever. Laboratory examinations revealed an increased WBC count of 16.7 × 10^3^ cells/μL and increased CRP concentration of 11.61 mg/dL. The tumor marker concentrations were within normal ranges, with a carbohydrate antigen 19-9 concentration of 4.7 ng/mL and a carcinoembryonic antigen concentration of 1.6 ng/mL. Ultrasound (US) showed a heterogeneous, mosaic-like echoic lesion, which was atypical for an abdominal abscess (**Fig. 1A**). Enhanced CT showed an 8-cm low-density tumor in the left side of the abdominal wall that was in contact with the small intestine (**Fig. 1B** and **1C**); these findings strongly suggested a malignant tumor, such as a sarcoma or metastatic tumor. Dynamic MRI revealed that the tumor had high signal intensity on T2-weighted images (**Fig. 1D**), which suggested that the mass was more likely to be an abscess than a malignant tumor. Positron emission tomography (PET)-CT showed fluorodeoxyglucose uptake corresponding to the solid component of the mass. The mass was suspected to have spread into the abdominal cavity, a finding suggestive of malignancy (**Fig. 1E**).

**Fig. 2:**
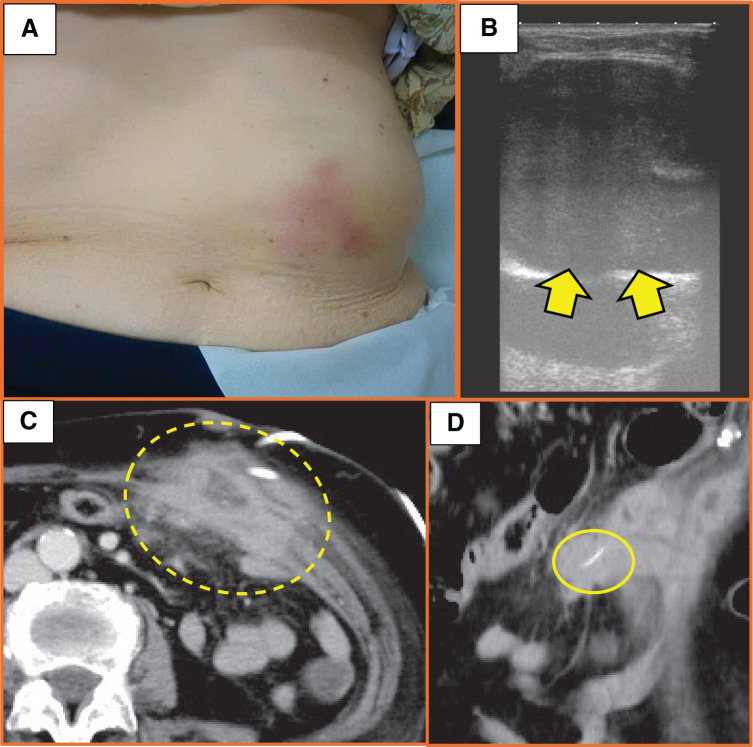
Physical findings and ultrasound 1 week after the first hospital visit, and enhanced CT images obtained on the 11th day after drainage. (**A**) The patient developed redness over the palpable mass. (**B**) Ultrasound shows increased fluid content in the lesion (yellow arrows). (**C**) Horizontal image shows that the low-density tumor shrank after abscess drainage (yellow dotted circle). (**D**) Sagittal image shows a new linear high-density structure (yellow circle).

One week after 1st visiting the hospital, the patient developed redness over the palpable mass (**Fig. 2A**), and laboratory examinations revealed further increases in the WBC count and CRP concentration. The US showed increased fluid content in the lesion (**Fig. 2B**). We then punctured the mass, and a gray liquid with a fecal odor emerged. A tumor biopsy was performed at the same time. The fluid suggested that the lesion might represent an abdominal wall tumor penetrating the small intestine. On the 11th day after drainage, follow-up CT showed that the mass had shrunk and contained a linear high-density structure (**Fig. 2C** and **2D**). Surprisingly, the biopsy results showed no malignant findings. At this point, our diagnosis was abdominal wall abscess perforating the small intestine, and we also suspected possible involvement of a fish bone. Considering the possibility that the abdominal wall tumor had perforated the small intestine, a plastic surgeon was called on standby and a surgical procedure was performed.

**Fig. 3:**
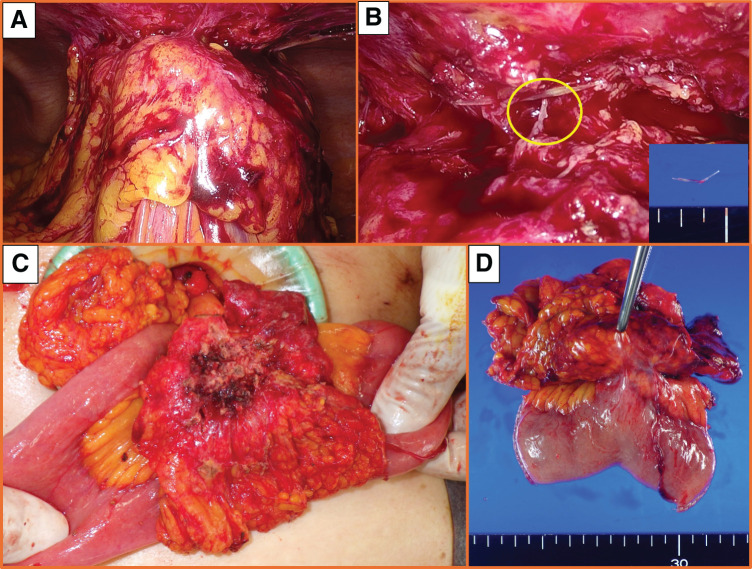
Surgical findings. (**A**) The small intestine and omentum are in a mass, and there are strong adhesions between the mass and the abdominal wall. (**B**) There is a 2-cm-long fish bone. (**C**) The abdominal wall abscess involves the small intestine, which has been partially resected. (**D**) The resected specimen shows the connection between the small intestine and the abscess.

Inspection of the peritoneal cavity with a laparoscope revealed that the small intestine and omentum had formed a mass with strong adhesions between the mass and the abdominal wall (**Fig. 3A**). We dissected the adhesions and removed a fish bone that was about 2 cm in length (**Fig. 3B**). We then extended the incision and performed partial resection of the small intestine, including the abdominal wall abscess (**Fig. 3C** and **3D**). The operation time was 2 h and 24 min, and the intraoperative blood loss was 40 g. The postoperative course was uneventful and the patient was discharged on postoperative day 9. The pathological findings of the specimen revealed no malignancy. The final diagnosis was an abdominal wall abscess caused by penetration of the small intestine by an ingested fish bone.

## DISCUSSION

It is fairly common to ingest a foreign body, such as a fish bone, and in most cases, the foreign body passes through the gastrointestinal tract without symptoms and does not require medical intervention.^4)^ However, in some cases, the ingested foreign body perforates or penetrates the gastrointestinal tract wall, causing peritonitis or an abscess.^5)^ In cases of abscess formation caused by an ingested fish bone, intra-abdominal abscesses are most common, while the formation of abdominal wall abscesses is exceedingly rare.^6)^ In the present case, the ingested fish bone penetrated the small intestine and was covered by the omentum, resulting in the formation of an abscess in the left abdominal wall. The diagnosis was delayed because of the difficulty in differentiating the abscess from inflammatory diseases or malignancy.

The primary reason for the difficulty in differentiating the abscess from malignancy in the present case was the similarity in clinical symptoms and imaging findings. Mass formation and abdominal wall abscesses are often associated with the progression of malignancy, and invasion of the abdominal wall suggests malignancy.^7)^ Additionally, the diagnosis was complicated by the patient’s lack of recollection of fish bone ingestion. However, many patients do not recall ingesting fish bones, making it difficult to suspect foreign body ingestion based on the clinical history.^8)^

In the present case, the mass was hard and poorly mobile, and the US did not show a clearly defined hypoechoic area typically seen in abscesses. In addition, PET-CT also raised suspicion of malignancy. Therefore, it was challenging to differentiate the abscess from malignant tumors, such as sarcoma. However, it is important to differentiate between sarcoma and abdominal wall abscess for appropriate surgical planning. On CT and MRI, sarcomas, such as leiomyosarcomas, typically present as a heterogeneous soft-tissue mass that often contains areas of necrosis, hemorrhage, or degeneration, with irregular enhancement of solid components.^9)^ Furthermore, sarcomas may invade the surrounding tissues, resulting in indistinct margins on imaging. In contrast, abdominal wall abscesses on CT and MRI are characterized as thick-walled fluid collections with peripheral ring enhancement that often contain gas with adjacent fat stranding.^10)^ On MRI, abscesses often show diffusion restriction due to the presence of purulent content, making them easier to distinguish from solid tumors.^11)^

The treatment of muscle abscesses is surgical drainage and antibiotics. Small abscesses can be treated with antibiotics alone but generally require early drainage.^12)^ In contrast, the main treatment for sarcoma is wide excision to reduce the risk of recurrence, as the surgical margins affect local control and survival rates. Wide excision for sarcoma in the abdominal wall often requires reconstruction.^13,14)^ In the present case, we suspected an abscess rather than a malignant tumor such as a sarcoma due to the reduction in the size of the mass observed after percutaneous drainage and the absence of malignant findings on biopsy. Therefore, we initially inspected the peritoneal cavity using a laparoscope. However, as malignancy could not be completely ruled out preoperatively, we had plastic surgeons available in case there was a need for abdominal wall reconstruction.

## CONCLUSIONS

We report a patient who had an abdominal wall abscess caused by penetration of the small intestine by an ingested fish bone and who underwent laparoscopic-assisted surgery. Such cases can be very difficult to diagnose preoperatively and require appropriate examinations and preparations before beginning treatment.

## ACKNOWLEDGMENTS

We thank Kelly Zammit, BVSc, from Edanz (https://jp.edanz.com/ac) for editing a draft of this manuscript.

## DECLARATIONS

### Funding

The authors declare that no funding was received for this case report.

### Authors’ contributions

TK participated in the writing of the manuscript.

SI and TK performed the operation.

TB and MI participated in drafting the work and revising critically for important intellectual content.

TU participated in review of the manuscript and final approval.

All authors read and approved the final manuscript and agreed to be accountable for all aspects of the work.

### Availability of data and materials

All data generated or analyzed during this study are included in this published article.

### Ethics approval and consent to participate

Not applicable.

### Consent for publication

Informed consent to publish the details of the case was obtained from this patient.

### Competing interests

The authors declare that they have no competing interests.
